# Tumor-Microenvironment Characterization of the MB49 Non-Muscle-Invasive Bladder-Cancer Orthotopic Model towards New Therapeutic Strategies

**DOI:** 10.3390/ijms24010123

**Published:** 2022-12-21

**Authors:** Sonia Domingos-Pereira, Karthik Sathiyanadan, Lenka Polak, Jacques-Antoine Haefliger, Martina Schmittnaegel, Carola H. Ries, Patrice Jichlinski, Beat Roth, Laurent Derré, Denise Nardelli-Haefliger

**Affiliations:** 1 Department of Urology, Lausanne University Hospital, University of Lausanne, 1010 Lausanne, Switzerland; 2 Department of Medicine, Lausanne University Hospital, University of Lausanne, 1010 Lausanne, Switzerland; 3 Roche Innovation Center Munich, Pharma Research and Early Development, 82377 Penzberg, Germany

**Keywords:** non-muscle-invasive bladder cancer, orthotopic MB49-bladder model, immune infiltration, chemokine expression, chemokine-targeting

## Abstract

Bacillus Calmette-Guérin (BCG) instillations for the treatment of non-muscle-invasive bladder cancer patients can result in significant side effects and treatment failure. Immune checkpoint blockade and/or decreasing tumor-infiltrating myeloid suppressor cells may be alternative or complementary treatments. Here, we have characterized immune cell infiltration and chemoattractant molecules in mouse orthotopic MB49 bladder tumors. Our data show a 100-fold increase in CD45^+^ immune cells from day 5 to day 9 tumors including T cells and mainly myeloid cells. Both monocytic myeloid-derived suppressor-cells (M-MDSC) and polymorphonuclear (PMN)-MDSC were strongly increased in day 9 tumors, with PMN-MDSC representing ca. 70% of the myeloid cells in day 12 tumors, while tumor associated macrophages (TAM) were only modestly increased. The kinetic of PD-L1 tumor expression correlated with published data from patients with PD-L1 expressing bladder tumors and with efficacy of anti-PD-1 treatment, further validating the orthotopic MB49 bladder-tumor model as suitable for designing novel therapeutic strategies. Comparison of chemoattractants expression during MB49 bladder tumors grow highlighted CCL8 and CCL12 (CCR2-ligands), CCL9 and CCL6 (CCR-1-ligands), CXCL2 and CXCL5 (CXCR2-ligands), CXCL12 (CXCR4-ligand) and antagonist of C5/C5a as potential targets to decrease myeloid suppressive cells. Data obtained with a single CCR2 inhibitor however showed that the complex chemokine crosstalk would require targeting multiple chemokines for anti-tumor efficacy.

## 1. Introduction

Bladder cancer (BCa) is the 10th most common malignancy, with more than 500,000 new cases diagnosed each year [1]. About 70% of BCa are diagnosed as non-muscle-invasive (NMIBC) [2] and according to specific tumor-stage and grade characteristics; intravesical immunotherapy with Bacillus Calmette-Guerin (BCG) is used to prevent recurrence and/or progression [3]. However, treatment failure may occur in 30–40% of cases [4,5], emphasizing the necessity to evaluate alternative or complementary therapies.

Among the numerous regulatory mechanisms generated during bladder tumor development [6], the presence of immune checkpoints, as well as myeloid regulatory cell subsets, have emerged as promising targets in BCa. Thus, blockade of PD-1/PD-L1 axis recently led to breakthrough clinical successes in muscle invasive BCa and several trials are ongoing in NMIBC [7]. In addition, the local balance between T lymphocytes and myeloid derived suppressor-cells (MDSC) may be predictive of BCG failure in the patients [8]. Decreasing the numbers of infiltrating MDSC and tumor associated macrophages (TAM) in bladder tumors, alone or in conjunction with BCG therapy, may also diminish recurrence and progression rates in NMIBC patients.

Here, we will focus on the targeting of TAM and/or MDSC chemoattractant axes, as it has become an attractive way to decrease those immunosuppressive cells. The chemoattractant CCL2 molecule has been involved in several cancers for inducing MDSC/TAM tumor infiltration and targeting this chemokine with anti-CCL2 antibodies and/or inhibitors of its major ligand, CCR2, have improved tumor regression in pre-clinical models of several cancers [9,10,11]. Although CCL2 was associated with poor survival in muscle-invasive or metastatic BCa [12], the CCL2-CCR2 axis was not previously targeted in NMIBC patients or in animal models of NMIBC. Other chemokine targets includes CCL20-CCR6 [13], CCL5-CCR5 [14] and/or CXCL12-CXCR4 [15] signaling which have been associated with TAM accumulation in breast cancer or glioma [10]. Chemokine receptors CXCR1/CXCR2 and their ligands (CXCL1-3, 5, 8) have been associated with tumor associated neutrophil (TAN) and/or polymorphonuclear–MDSC (PMN-MDSC) infiltration in various tumors [16], including BCa [17], and targeted in patients with breast cancer and prostate cancer [16].

Here, to design chemoattractant targeting, we have selected an orthotopic murine model, where syngeneic bladder tumor cells (MB49, a tumor cell line derived from a chemically induced urothelial carcinoma in a male C57BL/6 mouse [18]) are intravesically instilled, so that tumor deposition and seeding onto the mouse urothelium more closely reproduces NMIBC of patients. We characterized the immune microenvironment of growing MB49 bladder tumors including the kinetic of TAM and MDSC tumor infiltration, but also of the expression of the immune checkpoint PD-1/PD-L1. The kinetic of PD-L1 tumor expression correlated to the efficacy of an anti-PD-1 treatment in agreement with published results obtained in patients with PD-L1 expressing bladder tumors [19], thus suggesting that data obtained in the MB49 bladder tumor model may be informative for future therapeutic strategies. Our chemokine analysis highlighted the chemoattractants that may be targeted to decrease the immune myeloid suppressive cells in NMIBC, but also revealed a complex chemokine crosstalk, which deserves further attention to design novel anti-tumor-treatments.

## 2. Results and Discussion

### 2.1. Characterization of the Immune Cell Infiltration in MB49 Bladder Tumors and Model Evaluation

**Figure 1:**
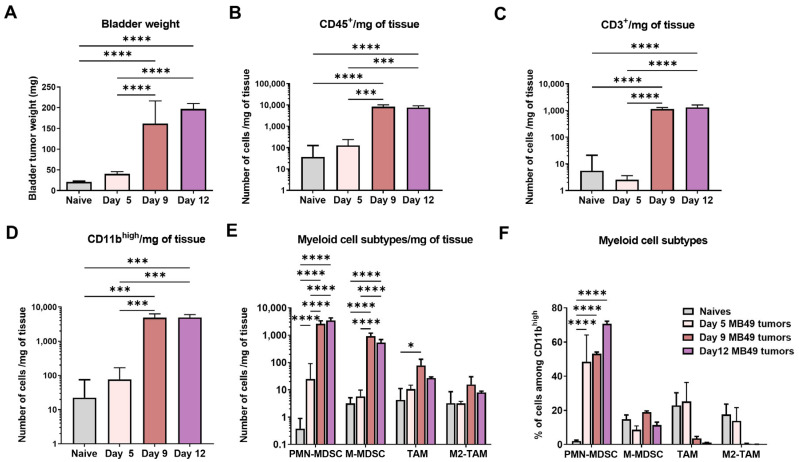
**Characterization of the immune infiltration in MB49 bladder tumors.** MB49-luc cells were intravesically instilled in the mouse bladder at day 1. Mice were then sacrificed at different time points (day 5 (*n* = 10), day 9 (*n* = 4) or day 12 (*n* = 4)). Naïve mice (*n* = 4) are shown as control. Bladder weight at each time point (mean ± SEM) is shown (**A**). Tissue-infiltrating immune cells were analyzed by flow cytometry. Absolute numbers per mg of tissues of CD45^+^ cells (**B**), T cells (CD3^+^, (**C**)), myeloid cells (CD11b^high^, (**D**)) and myeloid cell subtypes segregated as PMN-MDSC (Cd11b^high^Ly6G^high^), M-MDSC (CD11b^high^Ly6C^high^), TAM (CD11b^high^Ly6C^low^Ly6G^low^F480^+^ and M2-TAM (CD11b^high^Ly6C^low^Ly6G^low^F480^+^CD206^+^) (**E**). Myeloid cell subtypes are shown as percentages among CD11b^high^ cells (**F**). Significant differences are shown after one-way ANOVA followed by a Tukey’s comparison test **** = *p* < 0.0001 or *** = *p* < 0.001 or * = *p* < 0.05.

Immune cell infiltration from naïve bladder to growing MB49-bladder tumors (day 5, 9 and 12, Figure 1A) was examined after antibody staining of recovered cells by flow-cytometry (Supplementary Figure S1). A significant 100-fold increase of CD45^+^ immune cells/mg of tumor was observed from day 5 to day 9 tumors (Figure 1B), including CD3^+^ T-cells (Figure 1C), and myeloid-cells (CD11b^high^, Figure 1D). Myeloid-cell subtypes [20] (Figure 1E) included CD11b^high^Ly6G^high^ (a phenotype of PMN-MDSC), CD11b^high^Ly6C^high^ (a phenotype of M-MDSC), and CD11b^high^Ly6C^low^Ly6G^low^F480^+^ (a phenotype of TAM), with half of these TAM being CD206^+^ (a phenotype of immunosuppressive M2 TAM). In day 5 MB49 tumors a first significant influx of PMN-MDSC was observed (*p* < 0.001), while a significantly greater increase (ca. 100-fold, *p* < 0.001) of both M-MDSC and PMN-MDSC occurred in day 9 tumors. In contrast, TAM (ca. half of them being M2 TAM in naïve and in day 5 tumors) were only modestly increased at day 9 (ca. 5-fold, *p* < 0.05) and only represented <5% of the myeloid cell subtypes, which were dominated by PMN-MDSC (>70% at day 12, Figure 1F). Noteworthy, a positive correlation between PMN-MDSC infiltration and tumor size was also found in patients with BCa [21]. PMN-MDSC as the predominant type of MDSC was also previously reported in mouse models of thymoma, mammary carcinoma, melanoma, colon cancer and hepatocellular carcinoma, often associated with CCL2 and/or CCL9 chemoattractants [22,23].

**Figure 2:**
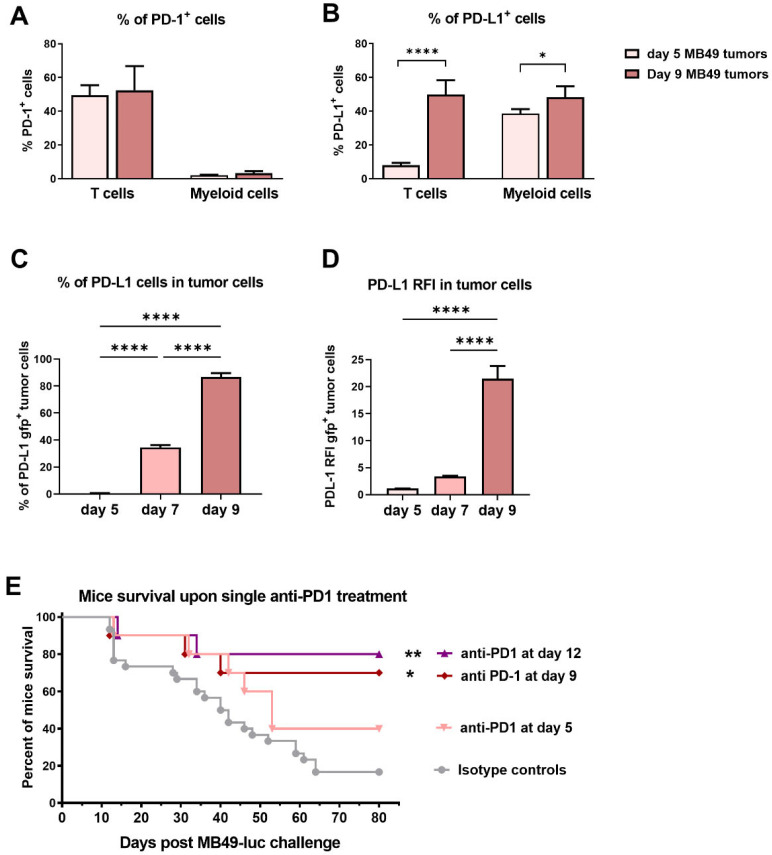
**Expression of the immune checkpoint PD-1 and PD-L1 and anti-PD-1 treatment efficacy.** MB49-luc cells (**A**,**B**,**E**) or MB49-gfp cells (**C**,**D**) were intravesically instilled in the mouse bladder. Mice were then sacrificed at different time points day 5 (*n* = 4), day 9 (*n* = 4) for (**A**,**B**) and day 5 (*n* = 4), day 7 (*n* = 5), day 9 (*n* = 5) for (**C**,**D**). The percentages of PD-1 (**A**) or PD-L1 (**B**) expressing T-cells or myeloid cells are shown. Percentage of PD-L1 expressing tumor cells (**C**) or relative fold increase (RFI) of PD-L1 expression (**D**) are shown. Mouse survival of groups of tumor-bearing mice (*n* = 10/group) that had received a single dose of 200 μg of an anti-PD-1 mAb at day 5, at day 9 or at day 12 or an isotype control are shown in (**E**). Significant differences are shown following one-way ANOVA followed by a Tukey’s comparison test (**A**–**D**) or an adjusted log rank test (**E**) **** = *p* < 0.0001, ** = *p* < 0.01 or * = *p* < 0.05.

Expression of the PD-1 and PD-L1 immune checkpoint receptor and ligand were examined in day 5 and day 9 MB49 tumors. PD-1 was already expressed in >50% of T cells and in a minority of myeloid cells (Figure 2A and Supplementary Figure S2A), while PD-L1 expression was only expressed in myeloid cell at day 5, but significantly increased at day 9 in both T cells (ca. 50%) and myeloid cells (ca. 50%, Figure 2B and Supplementary Figure S2B). To characterize the kinetic of PD-L1 expression on tumor cells, MB49 cells expressing the green fluorescent protein (gfp) were used for intravesical tumor implantation (Figure 2C,D and Supplementary Figure S2C). Data showed no detectable expression of PD-L1 in day 5 gfp+ tumor cells, followed by a progressive and significant increase of both percentage of cells and intensity of expression reaching a relative fold increase (RFI) of ca. 20-fold in day 9 tumors (*p* < 0.0001, Figure 2D). Accordingly, a single anti-PD-1 i.p. injection (200 μg/mouse) performed in MB49-tumor bearing mice at day 9 or at day 12 was sufficient to provide a significant anti-tumor efficacy with >70% survival at long term, while a single treatment at day 5 did not significantly improve mouse survival (Figure 2E), suggesting tumor expression of PD-L1 was necessary for anti-tumor efficacy. A similar and significant mouse survival of 70–80% was also obtained in our model after four consecutive intravesical instillations of BCG (1 week-apart, starting 1 day after tumor implantations [24,25], while a single BCG instillation at day 5 was ineffective [24] thus highlighting the efficacy of the single dose anti-PD-1 treatment. Of note, a significant, although lower, survival rate was previously reported with an anti-PD-L1 treatment performed from day 9 to day 15, in a similar orthotopic MB49 tumor model [26]. More importantly, our data are in agreement with efficacy results obtained in patients with PD-L1 positive, but not PD-L1 negative, bladder tumors [19], suggesting that the data obtained in the MB49 bladder tumor model may be informative for designing future therapeutic strategies.

### 2.2. Chemokine Expression in Growing MB49 Bladder Tumors

Chemokines levels in naïve bladder tissues and in growing MB49 bladder tumors (day 5, day 9 and day 15 tumors) were examined using a chemokine array including 25 chemokines. 18 chemokines were detected in naïve bladder comprising CCL2, CCL6, CCL8, CCL9/10, CCL12, CCL21, CCL22, CCL27, C5/C5a, Chemerin, CXCL1, CXCL5, CXCL9, CXCL11, CXCL12, CXCL16, CX3CL1 and IL-16 (Table 1). Significant increases in chemokines responsible for monocytes attraction (CCL6, CCL8, CCL9/10 and CCL12) and T-cells (CXCL9 and IL-16) were observed in day 5 bladder tumors, while chemokines responsible for neutrophils/PMN-MDSC and M-MDSC attraction (CXCL2, CXCL5, CXCL12 and C5/C5a) were further increased in larger day 15 tumors, together with additional T-cell chemoattractants (CCL5, CCL21, and CXCL10), in agreement with the kinetic of immune cell type infiltration in the tumors (Figure 1B–E). The higher increases (ca. 3–4 fold) were observed for CCL9/10 and CCL6 (murine chemokines, CCR1 ligands [27]), those levels then remained stable between day 5 and day 15 bladder tumors.

**Figure 3:**
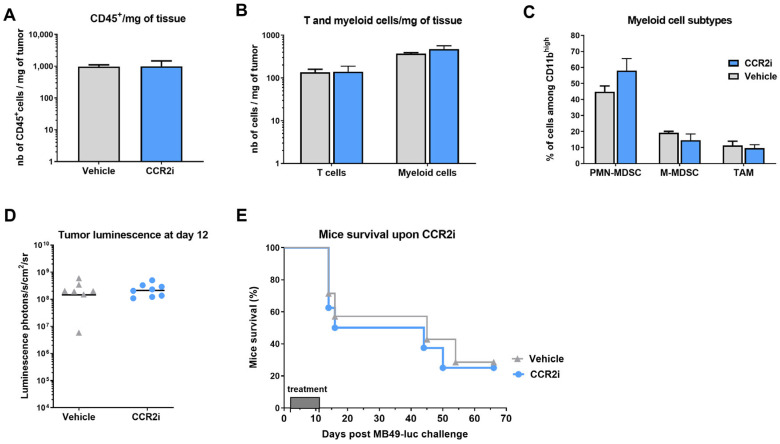
**CCR2i treatment of MB49-luc tumor bearing mice.** Mice bearing intravesical MB49-luc tumors were subcutaneously injected with 2 mg of a CCR2i twice a day for 10 days, starting 24 h after tumor instillation. A subset of 7 mice (vehicle *n* = 3, CCR2i *n* = 4) were sacrificed at day 9 for flow cytometry analysis (**A**–**C**). Absolute number/ mg of tumors of CD45^+^ cells (**A**), T cells and myeloid cells (**B**) are shown. The different myeloid subsets were further segregated (**C**) as PMN-MDSC (Cd11b^high^Ly6G^high^), M-MDSC (CD11b^high^Ly6C^high^), TAM (CD11b^high^Ly6C^low^Ly6G^low^F480^+^), and shown among CD11b^high^ cells. Tumor growth is shown by bioluminescence at day 12 in (**D**) and mice survival in (**E**) (vehicle *n* = 7, CCR2i *n* = 8). No significant differences were observed following a *t*-test (**A**–**D**) or an adjusted log rank test (**E**).

CCL9 was previously shown to recruit MDSCs in intestinal tumors [28] and hepatocellular carcinoma [29]. CCL6 has recently been shown to be secreted by eosinophil to promote metastatic tumor growth [30]. Pro-tumorigenic eosinophil have been associated with BCa [31] and whether the human CCL6 homologue, CCL23 [30], may be targeted to decrease pro-tumorigenic eosinophils deserve further investigation. The increase of the CXCR2 ligands CXCL2 and CXCL5, as well as of the CXCR4 ligand CXCL12 in day 15 MB49 bladder tumors is in agreement with their higher expression in BCa patients correlating with a poor prognosis associated with MDSC recruitment for CXCL2 [17] or to higher tumor cell migration/invasiveness for CXCL5 and CXCL12 [32,33,34]. The greater increase of complement C5/C5a in day 15 MB49 tumor, was also in agreement with higher expression of C5aR in BCa with poor prognosis [35]. The ability of C5a/C5aR blockade to decrease MDSC and tumor progression in a variety of cancers [36] thus deserve further investigation in bladder cancer. Interestingly, CCL2 chemokine levels did not vary during tumor growth in our NMIBC model, in contrast to increased levels of CCL8 and CCL12 (a murine chemokine) which are alternate ligands of CCR2 [23,27], suggesting that a CCR2 inhibitor (CCR2i) may decrease monocyte infiltration. We therefore tested in our MB49 bladder tumor model an available CCR2i that was previously shown to decrease TAM and inflammatory monocytes in a murine model of pancreatic cancer [37]. However, the data showed no significant alteration in immune cell infiltration (Figure 3A) neither for T or myeloid cells (Figure 3B), TAM or MDSC (Figure 3C), nor of tumor growth (Figure 3D) and mice survival (Figure 3E).

Chemokine analysis of MB49 tumors upon CCR2i treatment, as compared to control-treatment (Table 2), showed that some myeloid cell chemoattractants were significantly increased by CCR2i (C5/C5a, CCL11, CCL12, CCL8 and CX3CL1), while other were decreased (CXCL1, CXCL5, and CCL9/10), possibly explaining the status quo situation of the tumor immune cell infiltration.

The lack of effects observed in the MB49 bladder tumor model using a CCR2i differed from reports in pancreatic, liver, breast, prostate, renal, endometrial and ovarian cancer models [10,37,38], but may be in line with Fridlender et al., that reported that a CCL2 blockade was unable to reduce the influx of macrophages in s.c. human papillomavirus oncogene expressing TC-1 tumors, possibly due to other chemoattractants replacing CCL2/CCR2i axis [9]. This complex chemokine cross-talk thus suggest the necessity of targeting multiple chemokines for efficiently decreasing myeloid suppressor cells tumor infiltration. Of additional interest may also be the targeting of chemokines in combination with anti-PD-1/PDL1 treatment, which may however be stalled by the severe adverse events observed in recent clinical trials in pancreatic cancer (targeting CXCL12/CXCR4 + PD-1) and in colorectal, pancreatic, lung and hepatic cancer (targeting CCL2/CCR2 + PD-1) [39]. However, toxicity may be decreased by using the intravesical route of administration in NMIBC patients, as recently reported for selected patients receiving an anti-PD1 treatment [40], suggesting that other combination therapies may be similarly administered in NMIBC patients.

## 3. Conclusions

Our data point to the orthotopic MB49 BCa model as a suitable NMIBC model to design novel anti-tumor strategies. Chemokine analysis highlighted CCR2, CXCR2, CXCR4 and C5aR ligands/chemoattractants as possible targets to decrease the immune myeloid suppressive cells in NMIBC, but also revealed a complex chemokine crosstalk, which deserve particular attention to design novel anti-tumor-treatments.

## 4. Materials and Methods

***Tumor cell lines.*** The MB49 cell-line (kindly provided by Prof. A. Loskog, Uppsala University, Sweden) is derived from a carcinogen induced urothelial carcinoma in male C57Bl/6 mice [18]. Luciferase-expressing (MB49-luc) and green-fluorescent-expressing (MB49-gfp) cells were generated by transfection with lentiviral vectors encoding for firefly luciferase and gfp, respectively (kindly provided by Prof. D. Trono, EPFL, Lausanne, Switzerland).

***The MB49 orthotopic bladder tumor model.*** Seven to ten-week-old female C57BL/6 wild-type mice (Envigo, Gannat, france) were used and all experiments were performed in accordance with Swiss law and with approval of the Cantonal Veterinary Office of Canton de Vaud, Switzerland. Bladder tumors were established in deeply anesthetized mice that were urethrally catheterized using Introcan 24Gx3/4 catheters (Braun, Melsungen, Germany) as previously described [25]. A 15 min pre-treatment with 100 μL 22% ethanol was performed before instillation of 500,000 MB49-luc or MB49-gfp cells in 50 μL. MB49-luc tumor growth was monitored by bioluminescence 15 min after intraperitoneal (i.p.) injection of D-luciferin (Promega, Dübendorf, Switzerland, L8220, 150 μg/g of body weight) in the Xenogen imaging system (Xenogen/IVIS Caliper Life Science, kindly provided by cellular imaging facility, CIF/UNIL, Lausanne, Switzerland). 100% of the mice will develop bladder tumors and monitoring of MB49-luc tumors establishment and growth can be efficiently assessed during the first 3 weeks. Uncontrolled loss of luminescence of the growing tumors can then often appear [41], requiring additional monitoring by palpation, hematuria and overall health status of the mice, that were euthanized when they reached humane endpoints.

***Treatments.*** A single intraperitoneal injection of 200 μg of an anti-PD1 mAb (10 mg/kg, clone RPM1.14) or of its isotype control (an IgG2a, clone 2A3), both kindly provided by Roche, Penzberg, Germany, was performed at the indicated time points. CCR2 inhibitor (CCR2i, PF-413609, Med Chem Express, obtained from Roche, Penzberg, Germany, 10 mg/mL in 5% DMSO, 0.33% Tween80, in 0.9% NaCl.. Mice received 2 mg (100 mg/kg) subcutaneously in the flank, twice a day, starting 24 h after MB49 tumor cell instillation and for a duration of 10 days in tumor protection assays or until sacrifice for chemokine analysis. Control animals received similarly the vehicle alone.

***Immunostaining and flow cytometry analysis.*** Mice were sacrificed by CO_2_ inhalation to collect the bladders. Single-cell suspensions were obtained by mincing in DL-dithiothreitol (D9779, Sigma-Aldrich, Schaffhausen, Switzerland) and by subsequent digestion with 1 mg/mL collagenase/dispase (Roche, 11097113001) and 0.1 mg/mL DNAse I (D5025, Sigma-Aldrich, Schaffhausen, Switzerland) with 20% Fetal Calf Serum (10270, Gibco, Thermo Fisher Scientific, Waltham, MA, USA,). The recovered cells were stained and analyzed by flow cytometry. The following monoclonal anti-mouse antibodies were used: Anti-CD3-PE (17A2, 100206), Anti-CD3-PerCP/Cy5.5 (17A2, 100218), Anti-Ly6G-PE/Cy7 (1A8, 127618), Anti-CD11b-APC (M1/70, 101212), Anti-Ly6C-AF700 (HK1.4, 128024), Ly6C-APC/Cy7 (HK1.4, 128026), Anti-F4/80-APC-Cy7 (BM8, 123118), anti-CD206-PE (C068C2, 141706), anti-PD-1-PE (29F.1A12, 135205) from Biolegend, London, UK; Anti-CD45-PerCP/Cy5.5 (30-F11, 45-0451), anti-CD11b-eF450 (M1/70, 48-0112-82), anti-CD45FITC (30-F11, 11-0451-85), anti-CD274(PDL-1)-APC (MIH5-17-5982-80), anti-CD274(PDL-1)-PE (MIH5-12-5982-81) from eBioscience, Thermo Fisher Scientific, Whaltham, MA, USA). Dead cells were excluded by a live/dead fixable kit: aqua dead cell stain kit (L34957, Invitrogen, Thermo Fisher Scientific, Whaltham, MA, USA). Cell acquisition and analysis were performed using Gallios Flow Cytometer (Beckman Coulter, Nyon, Switzerland) and FlowJo 10.7.1 software (Tree Star, Ashland, OR, USA), respectively.

***Chemokine array.*** Bladders were homogenized in 500–1000 μL PBS with protease inhibitors (10 μg/mL Aprotinin from bovine lung, 10 μg/mL Leupeptin hemisulfate salt, and 10 μg/mL Pepstatin A; all from Sigma-Aldrich). TritonX-100 (final concentration 1%, Sigma-Aldrich) was added after homogenization, and after two freeze–thaw cycle’s samples were centrifuged at 10,000× *g* for 5 min to remove debris. Protein concentration was assessed using BCA protein assay (Thermo Fisher Scientific). Chemokines were detected using the Proteome profiler array: mouse chemokine array kit (R&D Systems), according to the manufacturer instructions. Briefly, 150 μg of protein (pooled from three bladders, 50 μg each) of each condition were used for the assay. Detection of chemokine levels was performed using ImageJ software (NIH, Bethesda, MD, USA) and expressed as mean pixel density. Significant detection was determined as > mean + 3SD of pixel density of negative spots. Increased chemokine levels between the different conditions and controls were considered significant when ≥ to the 99% confidence interval of the mean fold-increases examined.

***Statistics.*** Statistical analyses were performed using Prism 9.00 for Windows (GraphPad software, San diego, CA USA). Multiple comparisons were performed using one-way ANOVA and Tukey’s or Sidak post-test or log-rank test as indicated in the figure legends.

## Figures and Tables

**Table 1:** Chemokine levels in naïve bladder tissue and in growing MB49 bladder tumors.

Chemokine	Naïve Bladder (Mean Pixel Density ^a^)	Day 5 MB49 Bladder Tumor (Fold Change ^b^)	Day 9 MB49 Bladder Tumor (Fold Change ^b^)	Day 15 MB49 Bladder Tumor (Fold Change ^b^)
**CCL21**	17.6	1.6	1.6	**3.5**
**CXCL13**	ND ^c^	ND	ND	1.1
**CCL6**	35.4	**3.1**	**3.1**	**3.2**
**C5/C5a**	23.7	1.5	1.5	**3.4**
**CCL28**	ND	ND	ND	1.1
**Chemerin**	56.7	0.8	0.8	1.0
**CCL27**	17.4	1.1	1.1	1.2
**CXCL16**	14.2	1.5	1.5	1.4
**CCL11**	ND	ND	1.1	1.1
**CX3CL1**	14.0	ND	1.0	1.2
**IL-16**	30.1	**2.1**	**2.1**	**2.3**
**CXCL10**	ND	ND	**3.1**	**2.7**
**CXCL11**	13.3	ND	ND	1.1
**CCL2**	17.7	1.1	1.1	1.1
**CXCL1**	21.7	1.3	1.3	1.4
**CXCL5**	14.5	1.7	1.7	**2.8**
**CCL8**	18.0	**2.3**	**2.3**	**2.3**
**CCL12**	18.6	**1.8**	**1.8**	1.7
**CCL22**	13.4	ND	ND	1.2
**CXCL9**	13.3	**1.9**	**1.9**	**2.2**
**CCL3/CCL4**	ND	ND	ND	ND
**CCL9/10**	17.4	**3.6**	**3.6**	**4.0**
**CXCL2**	ND	ND	1.2	**2.1**
**CCL5**	ND	ND	1.3	**1.8**
**CXCL12**	19.3	1.2	1.2	**2.2**

^a^ chemokine levels are expressed as mean pixel density, when significantly higher than background signal + 3SD. ^b^ relative change in chemokine levels in day, 5, 9 or 15 bladder tumor as compared to naïve mice (when detectable) or to background signal + 3SD. Increased chemokine levels between naïve or background levels and day 5, 9 or 15 MB49 bladder tumors ≥1.8 (i.e., ≥ to the 99% confidence interval of the mean fold-increases) are indicated in bold. Chemokine increased between day 5 or 9 and day 15 are indicated in grey. ^c^ ND = not detectable.

**Table 2:** Chemokine levels in control mice and fold–changes upon CCR2i treatment in MB49 tumor bearing mice at day 9.

Chemokine	Control (Mean Pixel Density ^a^)	CCR2i (Fold Change ^b^)
**CCL21**	39.1	0.8
**CXCL13**	ND ^c^	ND
**CCL6**	170.3	1.0
**C5/C5a**	17.1	**1.4**
**CCL28**	ND	ND
**Chemerin**	40.5	0.8
**CCL27**	4.2	0.8
**CXCL16**	5.9	0.8
**CCL11**	3.8	**1.4**
**CX3CL1**	ND	**1.5**
**IL-16**	43.5	1.0
**CXCL10**	51.5	0.9
**CXCL11**	ND	ND
**CCL2**	16.5	0.8
**CXCL1**	10.8	**0.7**
**CXCL5**	7.8	**0.7**
**CCL8**	52.1	**1.3**
**CCL12**	31.5	**1.5**
**CCL22**	4.6	1.1
**CXCL9**	26.9	**0.6**
**CCL3/CCL4**	ND	ND
**CCL9/10**	104.8	**0.6**
**CXCL2**	ND	ND
**CCL5**	ND	ND
**CXCL12**	16.3	1.1

^a^ Chemokine level at day 9 in vehicle-treated MB49 tumor bearing mice (control) are expressed as mean pixel density, when significantly higher than background signal + 3SD. ^b^ relative change in chemokine levels (when detectable) after a CCR2i treatment as compared to vehicle-treated (control) mice. Increased/decreased chemokine levels between treatment and control ≥1.3 and ≤0.7 (i.e., ≤ and ≥ to the 99% confidence interval of the mean fold-increases) are indicated in bold. ^c^ ND = not detectable.

## Data Availability

Data is contained within the article or Supplementary Material.

## Supplementary Materials

The following supporting information can be downloaded at: https://www.mdpi.com/article/10.3390/ijms24010123/s1. Figure S1: Representative flow-cytometry plots showing the gating strategy in day 5 and day 9 MB49 tumor; Figure S2: Representative histograms of the immune checkpoint PD-1 and PD-L1 expression.

## References

[1] Bray F., Ferlay J., Soerjomataram I., Siegel R.L., Torre L.A., Jemal A. (2018). Global cancer statistics 2018: GLOBOCAN estimates of incidence and mortality worldwide for 36 cancers in 185 countries. CA A Cancer J. Clin..

[2] Babjuk M., Burger M., Zigeuner R., Shariat S.F., van Rhijn B.W., Comperat E., Sylvester R.J., Kaasinen E., Bohle A., Palou Redorta J. (2013). EAU guidelines on non-muscle-invasive urothelial carcinoma of the bladder: Update 2013. Eur. Urol..

[3] Babjuk M., Oosterlinck W., Sylvester R., Kaasinen E., Bohle A., Palou-Redorta J., Roupret M. (2011). EAU guidelines on non-muscle-invasive urothelial carcinoma of the bladder, the 2011 update. Eur. Urol..

[4] Yates D.R., Roupret M. (2011). Contemporary management of patients with high-risk non-muscle-invasive bladder cancer who fail intravesical BCG therapy. World J. Urol..

[5] Kamat A.M., Sylvester R.J., Bohle A., Palou J., Lamm D.L., Brausi M., Soloway M., Persad R., Buckley R., Colombel M. (2016). Definitions, End Points, and Clinical Trial Designs for Non-Muscle-Invasive Bladder Cancer: Recommendations From the International Bladder Cancer Group. J. Clin. Oncol. Off. J. Am. Soc. Clin. Oncol..

[6] Schneider A.K., Chevalier M.F., Derre L. (2019). The multifaceted immune regulation of bladder cancer. Nat. Rev. Urol..

[7] Roviello G., Catalano M., Santi R., Palmieri V.E., Vannini G., Galli I.C., Buttitta E., Villari D., Rossi V., Nesi G. (2021). Immune Checkpoint Inhibitors in Urothelial Bladder Cancer: State of the Art and Future Perspectives. Cancers.

[8] Chevalier M.F., Trabanelli S., Racle S., Cesson V., Gharbi D., Bohner P., Domingos-Pereira S., Dartiguenave F., Legris A.S., Speiser D. (2017). Local T-cell to MDSC balance is modulated by group-2 innate lymphoid cells and predictive of bladder cancer recurrence. J. Clin. Investig..

[9] Fridlender Z.G., Buchlis G., Kapoor V., Cheng G., Sun J., Singhal S., Crisanti M.C., Wang L.C., Heitjan D., Snyder L.A. (2010). CCL2 blockade augments cancer immunotherapy. Cancer Res..

[10] Argyle D., Kitamura T. (2018). Targeting Macrophage-Recruiting Chemokines as a Novel Therapeutic Strategy to Prevent the Progression of Solid Tumors. Front. Immunol..

[11] Iwamoto H., Izumi K., Mizokami A. (2020). Is the C-C Motif Ligand 2-C-C Chemokine Receptor 2 Axis a Promising Target for Cancer Therapy and Diagnosis?. Int. J. Mol. Sci..

[12] Amann B., Perabo F.G., Wirger A., Hugenschmidt H., Schultze-Seemann W. (1998). Urinary levels of monocyte chemo-attractant protein-1 correlate with tumour stage and grade in patients with bladder cancer. Br. J. Urol..

[13] Kadomoto S., Izumi K., Mizokami A. (2020). The CCL20-CCR6 Axis in Cancer Progression. Int. J. Mol. Sci..

[14] Aldinucci D., Borghese C., Casagrande N. (2020). The CCL5/CCR5 Axis in Cancer Progression. Cancers.

[15] Shi Y., Riese D.J., Shen J. (2020). The Role of the CXCL12/CXCR4/CXCR7 Chemokine Axis in Cancer. Front. Pharm..

[16] Susek K.H., Karvouni M., Alici E., Lundqvist A. (2018). The Role of CXC Chemokine Receptors 1-4 on Immune Cells in the Tumor Microenvironment. Front. Immunol..

[17] Zhang H., Ye Y.L., Li M.X., Ye S.B., Huang W.R., Cai T.T., He J., Peng J.Y., Duan T.H., Cui J. (2017). CXCL2/MIF-CXCR2 signaling promotes the recruitment of myeloid-derived suppressor cells and is correlated with prognosis in bladder cancer. Oncogene.

[18] Summerhayes I.C., Franks L.M. (1979). Effects of donor age on neoplastic transformation of adult mouse bladder epithelium in vitro. J. Natl. Cancer Inst..

[19] Massard C., Gordon M.S., Sharma S., Rafii S., Wainberg Z.A., Luke J., Curiel T.J., Colon-Otero G., Hamid O., Sanborn R.E. (2016). Safety and Efficacy of Durvalumab (MEDI4736), an Anti-Programmed Cell Death Ligand-1 Immune Checkpoint Inhibitor, in Patients With Advanced Urothelial Bladder Cancer. J. Clin. Oncol. Off. J. Am. Soc. Clin. Oncol..

[20] Kiss M., Van Gassen S., Movahedi K., Saeys Y., Laoui D. (2018). Myeloid cell heterogeneity in cancer: Not a single cell alike. Cell Immunol..

[21] Condamine T., Dominguez G.A., Youn J.I., Kossenkov A.V., Mony S., Alicea-Torres K., Tcyganov E., Hashimoto A., Nefedova Y., Lin C. (2016). Lectin-type oxidized LDL receptor-1 distinguishes population of human polymorphonuclear myeloid-derived suppressor cells in cancer patients. Sci. Immunol..

[22] Youn J.I., Nagaraj S., Collazo M., Gabrilovich D.I. (2008). Subsets of myeloid-derived suppressor cells in tumor-bearing mice. J. Immunol..

[23] Li B.H., Garstka M.A., Li Z.F. (2020). Chemokines and their receptors promoting the recruitment of myeloid-derived suppressor cells into the tumor. Mol. Immunol..

[24] Domingos-Pereira S., Cesson V., Chevalier M.F., Derre L., Jichlinski P., Nardelli-Haefliger D. (2017). Preclinical efficacy and safety of the Ty21a vaccine strain for intravesical immunotherapy of non-muscle-invasive bladder cancer. Oncoimmunology.

[25] Domingos-Pereira S., Sathiyanadan K., La Rosa S., Polak L., Chevalier M.F., Martel P., Hojeij R., Derre L., Haefliger J.A., Jichlinski P. (2019). Intravesical Ty21a Vaccine Promotes Dendritic Cells and T Cell-Mediated Tumor Regression in the MB49 Bladder Cancer Model. Cancer Immunol. Res..

[26] Vandeveer A.J., Fallon J.K., Tighe R., Sabzevari H., Schlom J., Greiner J.W. (2016). Systemic Immunotherapy of Non-Muscle Invasive Mouse Bladder Cancer with Avelumab, an Anti-PD-L1 Immune Checkpoint Inhibitor. Cancer Immunol. Res..

[27] Korbecki J., Kojder K., Siminska D., Bohatyrewicz R., Gutowska I., Chlubek D., Baranowska-Bosiacka I. (2020). CC Chemokines in a Tumor: A Review of Pro-Cancer and Anti-Cancer Properties of the Ligands of Receptors CCR1, CCR2, CCR3, and CCR4. Int. J. Mol. Sci..

[28] Kitamura T., Fujishita T., Loetscher P., Revesz L., Hashida H., Kizaka-Kondoh S., Aoki M., Taketo M.M. (2010). Inactivation of chemokine (C-C motif) receptor 1 (CCR1) suppresses colon cancer liver metastasis by blocking accumulation of immature myeloid cells in a mouse model. Proc. Natl. Acad. Sci. USA.

[29] Li B.H., Jiang W., Zhang S., Huang N., Sun J., Yang J., Li Z.F. (2020). The spleen contributes to the increase in PMN-MDSCs in orthotopic H22 hepatoma mice. Mol. Immunol..

[30] Li F., Du X., Lan F., Li N., Zhang C., Zhu C., Wang X., He Y., Shao Z., Chen H. (2021). Eosinophilic inflammation promotes CCL6-dependent metastatic tumor growth. Sci. Adv..

[31] Grisaru-Tal S., Itan M., Klion A.D., Munitz A. (2020). A new dawn for eosinophils in the tumour microenvironment. Nat. Rev. Cancer.

[32] Zheng J., Zhu X., Zhang J. (2014). CXCL5 knockdown expression inhibits human bladder cancer T24 cells proliferation and migration. Biochem. Biophys. Res. Commun..

[33] Gao Y., Guan Z., Chen J., Xie H., Yang Z., Fan J., Wang X., Li L. (2015). CXCL5/CXCR2 axis promotes bladder cancer cell migration and invasion by activating PI3K/AKT-induced upregulation of MMP2/MMP9. Int. J. Oncol..

[34] Yang D.L., Xin M.M., Wang J.S., Xu H.Y., Huo Q., Tang Z.R., Wang H.F. (2015). Chemokine receptor CXCR4 and its ligand CXCL12 expressions and clinical significance in bladder cancer. Genet. Mol. Res..

[35] Wada Y., Maeda Y., Kubo T., Kikuchi K., Eto M., Imamura T. (2016). C5a receptor expression is associated with poor prognosis in urothelial cell carcinoma patients treated with radical cystectomy or nephroureterectomy. Oncol. Lett..

[36] Senent Y., Tavira B., Pio R., Ajona D. (2022). The complement system as a regulator of tumor-promoting activities mediated by myeloid-derived suppressor cells. Cancer Lett..

[37] Sanford D.E., Belt B.A., Panni R.Z., Mayer A., Deshpande A.D., Carpenter D., Mitchem J.B., Plambeck-Suess S.M., Worley L.A., Goetz B.D. (2013). Inflammatory monocyte mobilization decreases patient survival in pancreatic cancer: A role for targeting the CCL2/CCR2 axis. Clin. Cancer Res..

[38] Mollica Poeta V., Massara M., Capucetti A., Bonecchi R. (2019). Chemokines and Chemokine Receptors: New Targets for Cancer Immunotherapy. Front. Immunol..

[39] Pu Y., Ji Q. (2022). Tumor-Associated Macrophages Regulate PD-1/PD-L1 Immunosuppression. Front. Immunol..

[40] Meghani K., Cooley L.F., Choy B., Kocherginsky M., Swaminathan S., Munir S.S., Svatek R.S., Kuzel T., Meeks J.J. (2022). First-in-human Intravesical Delivery of Pembrolizumab Identifies Immune Activation in Bladder Cancer Unresponsive to Bacillus Calmette-Guerin. Eur. Urol..

[41] Jurczok A., Fornara P., Soling A. (2008). Bioluminescence imaging to monitor bladder cancer cell adhesion in vivo: A new approach to optimize a syngeneic, orthotopic, murine bladder cancer model. BJU Int..

