# Supplementary figures and images for: Tumor-Microenvironment Characterization of the MB49 Non-Muscle-Invasive Bladder-Cancer Orthotopic Model towards New Therapeutic Strategies
Source: Int J Mol Sci. 2022 Dec 21;24(1):123. doi: 10.3390/ijms24010123 (PMC9820528; doi:10.3390/ijms24010123)

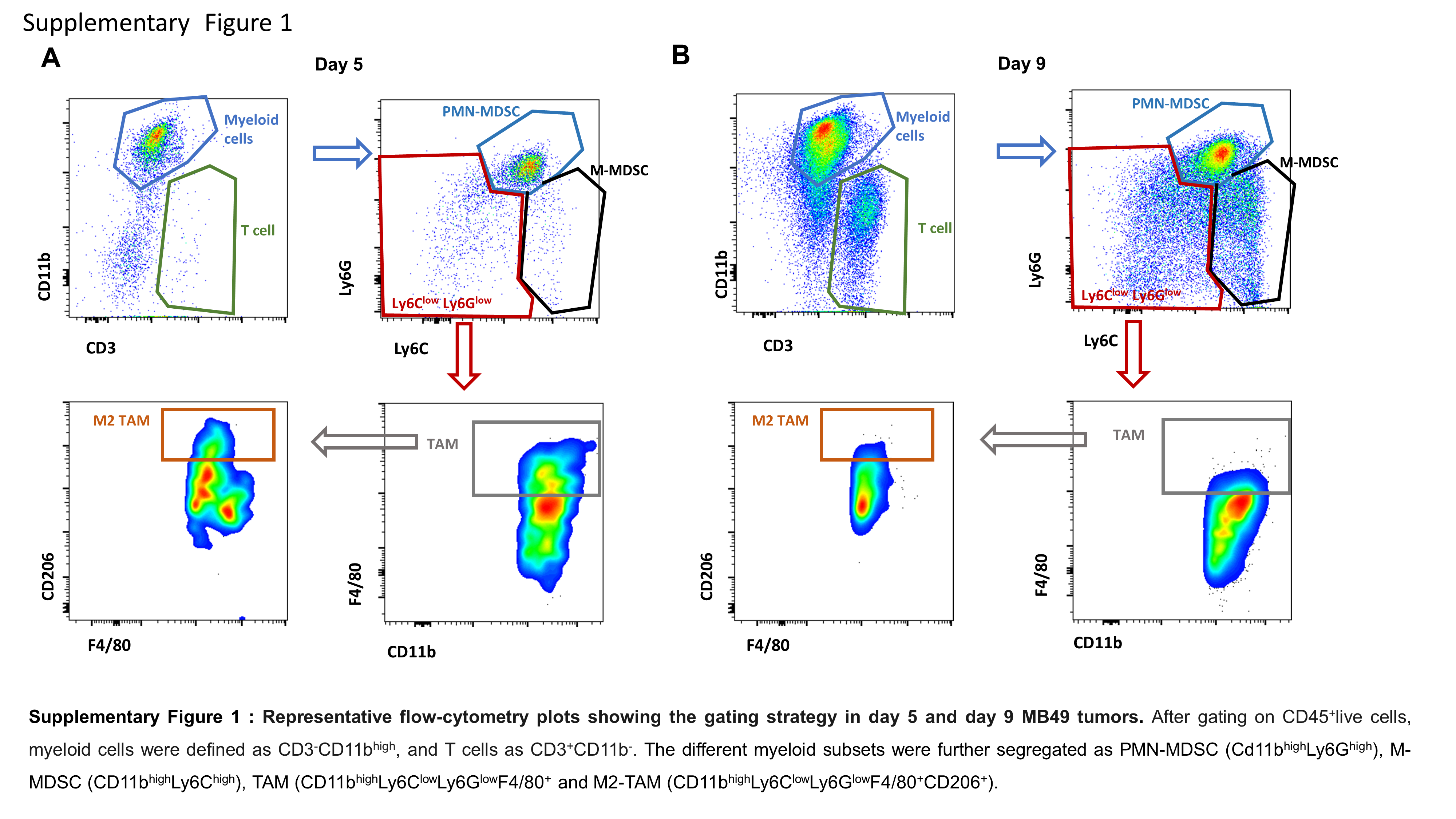

Supplement: Supplementary file 1 [file ijms-24-00123-s001.zip › Figure-S1-sup.TIF]

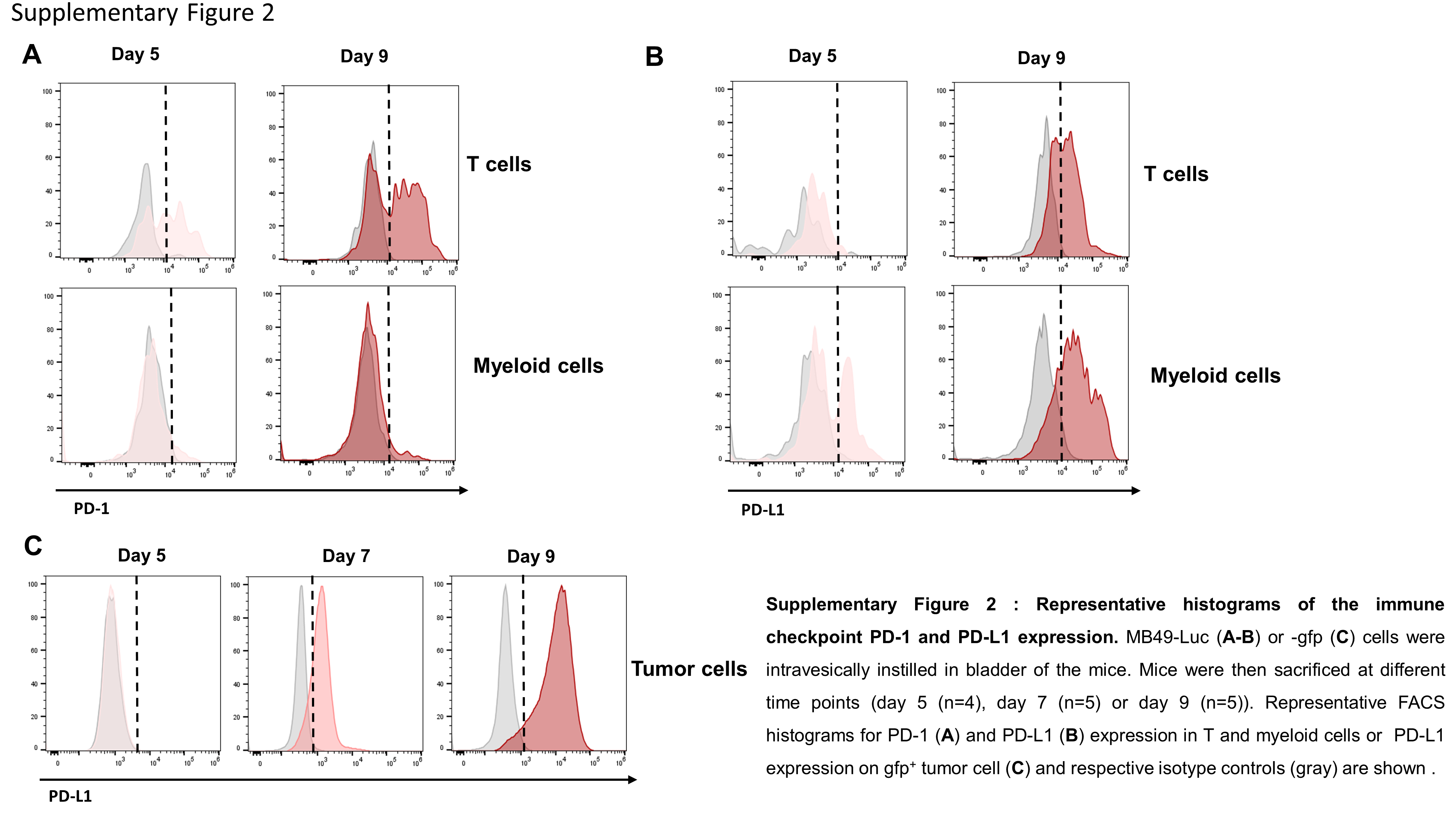

Supplement: Supplementary file 1 [file ijms-24-00123-s001.zip › Figure-S2-sup.TIF]
